# California Men's Health Study (CMHS): a multiethnic cohort in a managed care setting

**DOI:** 10.1186/1471-2458-6-172

**Published:** 2006-06-30

**Authors:** Shelley M Enger, Stephen K Van Den Eeden, Barbara Sternfeld, Ronald K Loo, Charles P Quesenberry, Sarah Rowell, Marianne C Sadler, Donna M Schaffer, Laurel A Habel, Bette J Caan

**Affiliations:** 1 Department of Research & Evaluation, Kaiser Permanente Medical Care Program, Pasadena, CA 91188, USA; 2 Division of Research, Kaiser Permanente Medical Care Program, Oakland, CA 94612, USA; 3 Department of Urology, Kaiser Permanente Medical Care Program, Bellflower Medical Center, Bellflower, CA 90706, USA; 4 Current at Kaiser Permanente Care Management Institute, Oakland, CA 94612, USA

## Abstract

**Background:**

We established a male, multiethnic cohort primarily to study prostate cancer etiology and secondarily to study the etiologies of other cancer and non-cancer conditions.

**Methods/Design:**

Eligible participants were 45-to-69 year old males who were members of a large, prepaid health plan in California. Participants completed two surveys on-line or on paper in 2002 – 2003. Survey content included demographics; family, medical, and cancer screening history; sexuality and sexual development; lifestyle (diet, physical activity, and smoking); prescription and non-prescription drugs; and herbal supplements. We linked study data with clinical data, including laboratory, hospitalization, and cancer data, from electronic health plan files.

We recruited 84,170 participants, approximately 40% from minority populations and over 5,000 who identified themselves as other than heterosexual. We observed a wide range of education (53% completed less than college) and income. PSA testing rates (75% overall) were highest among black participants. Body mass index (BMI) (median 27.2) was highest for blacks and Latinos and lowest for Asians, and showed 80.6% agreement with BMI from clinical data sources. The sensitivity and specificity can be assessed by comparing self-reported data, such as PSA testing, diabetes, and history of cancer, to health plan data. We anticipate that nearly 1,500 prostate cancer diagnoses will occur within five years of cohort inception.

**Discussion:**

A wide variety of epidemiologic, health services, and outcomes research utilizing a rich array of electronic, biological, and clinical resources is possible within this multiethnic cohort. The California Men's Health Study and other cohorts nested within comprehensive health delivery systems can make important contributions in the area of men's health.

## Background

In 2002 – 2003, we established the California Men's Health Study (CMHS), a multiethnic cohort of 84 170 men ages 45 to 69 years who are members of the northern and southern California regions of Kaiser Permanente, the largest managed care organization in California. The major goal of the study is to improve understanding of the development of prostate cancer due to the huge impact it exerts on morbidity and mortality of American males, especially African-American males [1]. By establishing the cohort within an integrated managed care organization, we have access to rich electronic data sources to supplement clinical data collected by survey and to permit the identification and investigation of other health outcomes including other cancers and other non-cancerous conditions.

We obtained baseline data from the men using a two-stage mailed survey, and we employed unique recruitment techniques to enrich the cohort with non-white men. With this cohort, we plan to build on previous work and investigate novel hypotheses in the broad area of men's health. This paper is to describe the baseline demographic characteristics, health status, and lifestyle behaviors of the cohort, to estimate expected cases of cancer, to evaluate the validity of the self-reported data, and to examine how representative the cohort was to the larger population.

## Methods/design

### Participant eligibility criteria and identification

Eligible participants included all male California Kaiser Permanente (KP) Health Plan members, aged 45 to 69 years in January 2000, who had been members of the health plan for at least one year at recruitment. We identified potential participants from electronic KP membership files containing each member's birthdate, current address, and membership history. At recruitment, nearly 850,000 health plan members met the eligibility criteria.

The study protocol was reviewed and approved for the protection of human subjects by the Institutional Review Boards of Kaiser Permanente Northern California and Kaiser Permanente Southern California.

### Participant recruitment and data collection

We recruited participants beginning in January 2002 and concluding at the end of December 2003 using a two-step process in three mailing waves. In the first step of each mailing wave, we mailed a recruitment letter and a short questionnaire in English/Cantonese to potentially eligible health plan members with Chinese surnames [2,3] or in English/Spanish to all other potentially eligible members. The short questionnaire requested information on the men's race/ethnicity, information not routinely collected by the health plan. In the second step of each mailing wave, we mailed a cover letter and a longer, comprehensive questionnaire to participants who had completed and returned the short questionnaire. The cover letter and questionnaire were in the language in which the participant completed the short questionnaire, and the cover letters accompanying the questionnaires were tailored to the participant's specific race/ethnic group and were signed by a well known athletic (black/African-American) or political (Latino) figure or by one of the study investigators (all others). Members were also offered the option to complete the questionnaires electronically in English on a secure website (NCS Pearson, Inc.).

Recruitment mailings were done in three waves using mailing strategies to enhance recruitment of minority male members. In mailing wave one, we mailed to members residing near KP medical centers serving the highest estimated proportion of African-American men and to members we identified with Chinese [2,3] Spanish surnames [4]. In mailing wave two, we mailed to men either who were listed as minority based on hospitalization information or who resided near additional KP facilities with high minority populations. In mailing wave three, due to limited study funds, remaining potentially eligible members were mailed a recruitment letter inviting them to complete both questionnaires on the website, and they had the option of requesting a paper questionnaire. Mailing waves included 200,000 to 350,000 members.

### Questionnaires

The short (2-page) questionnaire included questions on race/ethnicity, anthropometrics, prostate specific antigen (PSA) testing history and personal history of prostate cancer or benign prostatic hyperplasia (BPH).

The longer (24-page) questionnaire solicited information on demographics, family history of cancer, health and lifestyle, prostate-related symptoms and conditions, other existing health conditions, medication/drug use, physical activity, tobacco use, diet/supplement use, country of origin, duration of U.S. residency, income, and sexual development, orientation, and health. Lower urinary tract symptoms were assessed using the American Urology Association Symptom Index (AUASI) [5], while erectile dysfunction was assessed by a four-level single question adapted form the Massachusetts Male Aging Study [6]. We assessed diet with a detailed semi-quantitative food-frequency questionnaire adapted from a questionnaire developed for the Women's Health Initiative and other studies [7-9], modified to men's studies of prostate health [10]. We assessed physical activity with questions adapted from the CARDIA Physical Activity History [11-13] that queried the men about the frequency and duration of their participation in specific types of moderate and vigorous recreational, household, and work-related activities. The CARDIA questionnaire has indirect validity against aerobic capacity and percent body fat [11,12] and a strong inverse relation with most cardiovascular disease risk factors [14,15].

We manually reviewed paper questionnaires for stray marks, completeness, and multiple responses. We optically scanned the questionnaires into study computers using ScanTools^® ^II Software (Pearson NCS, Inc, Bloomington, MN). We uploaded the data into SAS data management databases (SAS Statistical Software Version 8.2, SAS Statistical Institute, Cary, NC) for cleaning and storage. We used computerized edits to assess the data for logic errors, out-of-range values, missing data, and multiple responses.

### Analytical/statistical methods

We constructed the variables reported in the tables from both self-reported and health plan data. We linked study databases with electronic health plan databases using the member's health record number, a unique subject identifier. The health plan data provided information about the participants' KP membership history, hospitalization history, laboratory results, and other health conditions including cancer and diabetes. We used health plan data to validate specific survey data items when comparable health plan data existed.

We created new variables and categorical variables from the raw questionnaire data. Body mass index (BMI), a measure of obesity, was calculated as weight (kilograms) divided by the square of height (meters^2^). Participants who did not complete all pages of the food frequency questionnaire (at least five items per page) or who had total calculated energy intake below (less than 800 kcal) or above (greater than 5,000 kcal) what we considered reasonable were excluded from the nutrient analyses. Recreational physical activity summary scores were derived by multiplying assigned MET values^13 ^by duration and frequency and summing across activities. We defined vigorous activities as participating in a minimum of 1,260 MET-hours per week, on average, equivalent to at least 3.5 hours of activity with a minimum MET level of 6. We defined moderate and vigorous activities combined as participating in a minimum of 630 MET-hours of activity per week, on average, equivalent to at least 3.5 hours of activity with a minimum MET level of 3.

We determined the expected number of cases of prostate, colorectal, lung, and bladder cancer and melanoma based on the expected baseline age distribution of the cohort of 84,170 men, and the age and race/ethnic specific annual (average 1992–1996) cancer incidence rates for the State of California. We account for an assumed 4% annual rate of loss to follow-up, due to mortality and loss to follow-up, based on our experience with other cohort studies.

We calculated percent agreement between BMI derived from self-reported weight and height and BMI from clinical databases. We cross tabulated the data grouped according to BMI categories of normal weight (<25), overweight (25–29), and obese (30 or higher), and then calculated the percent agreement as the total number of participants for whom BMI was classified in the same category by both sources divided by the total number of participants with clinical BMI data available.

### Cohort characteristics

Response rates varied across mailing strategies (Table 1). Short questionnaire responses were substantially higher when we mailed questionnaires (waves one and two) than when we invited the men to complete the questionnaire on-line (wave three). Conversely, among those who completed the short questionnaire, the responses to the long questionnaire were 50% higher among men recruited in wave three compared to men recruited in waves one and two. Overall, the participants completed nearly one-quarter of the short questionnaires and nearly one-third of the long questionnaires on the secure website.

A total of 84170 men joined the cohort, and nearly 40% were minority (Table 2). Participants were distributed fairly evenly across age groups, although Latino, Asian, and 'other/mixed race/ethnicity' men tended to be somewhat younger, a reflection of the general KP membership age distribution. Educational attainment varied by race/ethnicity, with over half of whites and Asians having earned at least a college degree compared to one-third of black participants and over one-fifth of Latinos. Income distributions also varied by race/ethnicity, with white participants reporting the highest and Latinos reporting the lowest incomes.

One in five participants had a history of BPH, a benign but frequently symptomatic prostate enlargement (Table 3). Rates among white men were the highest and were more than 50% higher than those of Asian men who experienced the lowest rates. A history of prostatitis, a painful inflammation of the prostate usually due to infection, was also most common among white participants and more than twice as common as among Asians. Nearly half (48.6%) of the cohort reported moderate or severe LUTS, while 29.9% of the men reporting sometimes or never being able to get or keep an erection (e.g., erectile dysfunction).

Most participants reported having received PSA testing sometime in the past (Table 3). Black participants were most likely and Latino participants were least likely to have reported receiving a test. We observed only slight race/ethnic variation in the percentage of participants who received fasting glucose, HDL cholesterol, cholesterol panel, or triglyceride laboratory tests within the five years before cohort enrollment. In general, however, Asian participants were most likely and Latino participants were least likely to have received the tests.

We observed that energy and nutrient intakes varied somewhat by race/ethnicity (Table 4). Asian participants reported the lowest consumption of calories and percent calories from fat, fiber, protein and alcohol. Conversely, white participants reported the highest consumption of calories, fiber, and alcohol. Fruit consumption (servings) was similar across race/ethnic groups.

The median BMI for the cohort was high with more than 50% of participants considered overweight (Table 4). Blacks and Latinos had the highest and Asians had the lowest median BMI. Nearly two-thirds of participants reported regular moderate or vigorous recreational activities and over 40% reported regular vigorous recreational activities, with the highest rates among white participants. Although smoking rates were low overall, rates among black participants, the highest, were more than double the rates of Asian participants, the lowest.

Over 5,000 participants identified themselves as other than heterosexual, with the highest proportion among white and the lowest proportion among Asian participants (Table 4).

We anticipate that nearly 1,500 prostate cancer diagnoses will occur among participants during the 5 years after cohort inception (Table 5). We also anticipate sufficient diagnoses of lung and colorectal cancers, and other diseases of similar incidence, within 5 years to conduct well powered analyses of etiological factors. There may also be sufficient bladder cancer and melanoma diagnoses to conduct basic etiological analyses.

In addition to cancer outcomes, a significant number of newly diagnosed cases of cardiovascular diseases and neurodegenerative diseases are expected. Based on estimates in the literature, estimates of the expected number of new cases in the CMHS participants after five years of follow-up (and adjusting for loss of follow-up and mortality) include: 5,000 new cases of myocardial infarction, 5,100 cases of congestive heart failure, 180 cases of Parkinson's disease, 7,000 cases of dementia, and 370 cases of Alzheimer's disease.

We compared CMHS participants with male participants in the California Health Interview Survey (CHIS), a population-based telephone survey of 55,000 California residents in 2001 [16] (Table 6). Distributions of participants were similar for the demographic factors examined as well as for BMI. Substantially more CMHS participants had reported at least one PSA test than CHIS participants, probably due to complete insurance coverage among CMHS participants.

We also performed a small comparison of CMHS participants to non-participants of the same age range. A total of 84.4% CMHS participants were KP members for at least five years compared to 75.3% of non-participants. The percentage hospitalized during the 5 years before recruitment were similar for the two groups (17.0% of participants versus 15.6% of non-participants), and the prevalence of diabetes mellitus was nearly identical (13.5% for participants versus 13.6% for non-participants).

We validated BMI calculated from self-reported weight and height in relation to BMI reported in health plan clinical databases (Table 7). Overall, the percentage of participants who were classified into the same BMI categories by both sources was over 80%, and the percent of participants who were classified into the same or adjacent categories was 99.4%.

## Discussion

We assembled a large, multiethnic male cohort from the membership of the largest managed care organization in California to study prostate cancer etiology and the etiologies of other cancer and non-cancerous conditions. CMHS participants are diverse in socioeconomic status as well as race/ethnicity, where both ends of the income and education spectrum are well represented in the cohort. The managed care setting is ideal to study disease risk factors and health services delivery where health care access issues are minimized and supplemental data sources are maximized and can be used to verify and extend the questionnaire data.

The availability of supplemental electronic data is a unique and important strength of this cohort. For example, we can conduct electronic data linkages between cohort and electronic health plan files to obtain current and historical data, obtain information on outcomes other than cancer, and conduct validation studies of self-reported data. Electronic data sources can be used to follow the participants for updated contact information from membership files, cancer diagnoses through cancer registry databases, and deaths of through linkage with health plan mortality databases derived from state death certificate files.

Another advantage of these data within this setting is the ability to assess the sensitivity and specificity of the self-reported data on health conditions and PSA testing by comparing these data to clinical databases of the health plan. The BMI from self-report was in high agreement with clinical BMI data, suggesting that data collection by self-report was reliable. However, a limitation of these analyses is the inability to identify diagnoses that pre-dated the participant's health plan membership or the inception date of the electronic database.

Another unique strength of this cohort is the potential to conduct efficient biospecimen-based research studies. The health plan maintains tissue banks of tumor and non-tumor tissue blocks, some from as early at the 1950s, and the health plan manages a system of clinical laboratories that can be used for the efficient collection of blood specimens. The availability of these resources greatly facilitates research of genetic markers and other molecular studies.

Prospective cohort studies are considered the gold standard of epidemiologic research because many biases that can hamper interpretation of case-control studies are, by design, minimized in prospective cohort studies [17,18]. Cohort studies also facilitate the study of multiple outcomes, unlike other observational study designs. A drawback to cohort studies is the long follow-up time often required to obtain sufficient power to conduct statistical analyses, typically low recruitment rates, and loss to follow-up that can harm internal validity. However, we anticipate having sufficient statistical power within five years of cohort inception to conduct prostate cancer analyses. Our cost-saving strategies of not re-contacting men who did not respond initially and of mailing the long questionnaire only to men who completed the 2-page questionnaire enhanced the likelihood of obtaining favorable follow-up rates and therefore high internal study validity, because the final cohort included only the men who were willing to complete two questionnaires separated in time.

The relatively low recruitment rates raise the issue of generalizability of the study findings to the broader community, although this aspect of this study is within the range that is found in other general cohort recruitment efforts. Prospective cohorts are considered valuable partly because well conducted and followed cohorts allow internally valid comparisons to be made [18-21], similar to clinical trials which are considered to be internally valid despite inclusion of typically highly selected participants [22]. No cohort is strictly generalizable to the population as a whole. Rather, research from some of the most widely cited cohorts is derived from study populations that are overwhelmingly from one race/ethnic group [23-30], occupational group or class group [23,24,26,27], or observational studies following clinical trials [31, 32, 33]. Nonetheless, the CMHS cohort was similar to the population of health plan members on important characteristics and appeared similar to men who responded a general health survey in California on a variety of important demographic and clinical characteristics. Thus, a cohort such as this one should be able to address biologically relevant questions related to disease onset and progression.

A wide variety of epidemiologic, health services, and outcomes research is possible within this multiethnic cohort. The cohort is available for study by non-Kaiser Permanente investigators and students conditional upon approval by the CMHS Proposal Review Committee. A rich array of electronic, biological, and clinical resources is available to supplement and enhance the survey data and to facilitate the study of outcomes other than cancer while controlling for major health risk factors. The large prospective cohorts in the United States have made enormous contributions to our knowledge of the causes of cancer and other diseases, and we believe the CMHS is poised to make important contributions in the area of men's health.

## Competing interests

The author(s) declare that they have no competing interests.

## Authors' contributions

SME was the principal author of the manuscript. SME, BS, SKV, RKL, CPQ, SR, DMS, and BJC made substantial contributions to the conception and design of the study, and to the acquisition of funding. SME, BS, SKV, RKL, CPQ, MCS, LAH and BJC contributed to the analysis of the data. All authors contributed to the interpretation of the data, the drafting of manuscript, and have read and given approval to the final version of the manuscript.

## Pre-publication history

The pre-publication history for this paper can be accessed here:



## Figures and Tables

**Table 1:** Recruitment figures: CMHS Cohort, California.

Mailing Wave	Total number Recruited	Number of responses	Response %
1 and 2 (paper with electronic option^1^)			
Short questionnaire	503 237	114 953	22.8
Long questionnaire	114 953	67 413	58.6
			
3 (electronic with paper option^2^)			
Short questionnaire	345 262	19 107	5.4
Long questionnaire	19 107	16 757	87.7
			
Total – 1, 2 and 3			
Short questionnaire	848 499	134 060	15.8
Paper		103 383	
Electronic		30 677	
Long questionnaire	134 060	84 170	62.8
Paper		57 204	
Electronic		26 966	

^1 ^Participants were mailed paper questionnaires but given the option to complete the questionnaires on a secure website

^2 ^Participants were invited to complete the questionnaires on a secure website but offered the option to request a paper questionnaire

**Table 2:** Distribution of participants by age, educational attainment, and current household income, by race/ethnicity: CMHS Cohort, California.

	Race/Ethnicity (%)						
	White N = 51 746	Black/African American N = 6302	Latino N = 11 428	Asian N = 6400	Other/mixed N = 7459	Missing N = 835	Total N = 84 170

Age (years)							
45–49	13.7	14.9	22.3	17.9	19.0	16.0	15.7
50–54	19.1	18.8	22.2	22.9	20.9	17.8	20.0
55–59	23.0	20.5	19.3	18.4	21.4	19.1	21.8
60–64	22.3	23.0	18.1	18.8	20.8	21.1	21.4
65–69	21.6	22.6	17.8	21.8	17.7	25.7	20.9
Educational Attainment							
Less than high school	2.6	5.7	24.4	6.5	4.4	9.0	6.3
HS grad or GED	10.7	16.3	18.5	9.6	11.7	16.7	12.2
Vocational/tech school	4.0	5.0	7.9	5.1	5.3	6.9	4.8
Some college/AA	29.7	39.8	27.7	20.3	32.6	31.3	29.8
College graduate	22.1	16.4	10.1	28.5	26.0	16.4	20.8
Grad/prof school	30.6	16.5	10.9	29.7	19.8	19.4	25.8
Current household income ($)							
Less than 20,000	3.3	6.2	10.9	6.3	5.5	8.5	5.0
20 000 – 39 999	12.6	18.7	27.8	15.5	17.0	22.7	15.8
40 000 – 59 999	18.2	21.9	22.5	19.2	20.7	21.8	19.4
60 000 – 79 999	18.7	19.9	17.2	16.8	19.6	19.0	18.5
80 000 – 99 999	14.7	13.4	9.5	14.2	13.5	10.5	13.7
100 000 or more	32.1	19.5	11.8	27.7	23.4	17.3	27.2

**Table 3:** Distribution of specific prostate conditions and laboratory tests, by race/ethnicity: CMHS Cohort, California.

	Race/ethnicity (%)					
	White	Black/African-American	Latino	Asian	Other	Total

Prostate diagnoses and procedures^1^						
BPH diagnosis	22.9	21.5	17.5	13.8	16.7	20.8
Prostatitis diagnosis	7.6	6.2	5.5	3.4	5.1	6.6
Laboratory tests^2^						
PSA	75.5	83.8	67.9	72.9	71.0	74.5
Fasting glucose	59.0	63.4	57.2	65.0	64.0	60.0
HDL cholesterol	55.0	50.5	45.8	66.1	58.1	54.5
Cholesterol panel	80.7	82.4	77.1	86.3	82.6	80.9
Triglycerides	68.0	66.8	63.6	71.3	70.3	67.8

^1 ^Prostate diagnoses and procedures were obtained from survey data where participants reported having ever been diagnosed with BPH or prostatitis

^2 ^Laboratory test data were obtained from electronic health plan databases and included tests done during the five-year period before cohort enrollment

**Table 4:** Mean daily energy and nutrient intakes, median body size, percentage of participation in moderate and vigorous physical activities, current smoking status, and sexual orientation, by race/ethnicity, based on self-reported survey data: CMHS Cohort, California.

	Race/Ethnicity					
	White	Black/African American	Latino	Asian	Other	Total

Dietary factors						
Calories	2218.3	2024.5	2037.1	1765.0	2088.6	2139.0
% calories from fat	34.8	35.5	34.8	32.8	34.6	34.7
Dietary fiber (gm)	22.3	18.3	21.5	17.2	20.1	21.4
Protein (gm)	95.9	87.1	88.8	84.1	93.0	93.3
Fruit (servings)	2.2	2.3	2.3	2.2	2.3	2.2
Vegetables (servings)	2.2	1.9	1.9	1.7	2.1	2.1
Alcohol (gm)	13.4	8.6	9.1	4.4	8.9	11.5
BMI (median)	27.2	28.2	28.2	24.7	26.7	27.2
Physical activity (%)						
Moderate/vigorous^2^	65.7	49.2	52.9	57.7	57.2	61.2
Vigorous^3^	46.6	33.5	36.2	37.4	39.1	42.8
Current smoker (%)	9.5	17.0	14.4	8.2	11.8	10.8
Sexual Orientation (%)						
Heterosexual	93.7	95.4	94.4	94.5	90.9	93.7
Homosexual	4.4	1.8	2.2	1.2	3.0	3.5
Bisexual	1.3	1.1	1.2	1.5	1.9	1.3
Other	0.5	1.5	2.1	2.6	4.0	1.2

^1 ^moderate or vigorous activity defined as a minimum of 630 MET-hours activity per week on average, equivalent to at least 3.5 hours of activity with MET level minimum of 3.

^2 ^vigorous activity defined as a minimum of 1260 MET-hours activity per week on average, equivalent to at least 3.5 hours of activity with MET level minimum of 6.

**Table 5:** Expected number of cancer diagnoses by December 31, 2006: CMHS Cohort, California.

	Cancer Site				
	Prostate	Colorectum	Lung	Skin: Melanoma	Bladder

White	933	284	449	133	183
Black/African American	203	46	85	1	11
Latino	131	43	41	4	13
Asian/Pacific Islander	77	45	51	1	11
Other	116	26	23	24	18
Total	1460	444	648	162	237

**Table 6:** Self-reported demographic, body mass index, and prostate cancer screening data in the CMHS Cohort compared to California Health Interview Survey* (CHIS), males aged 50 to 64 years.

	CMHS %	CHIS %
Race/Ethnicity		
White	63.3	63.9
Black/African-American	7.4	5.6
Latino	12.9	17.5
Asian	7.3	9.9
Other	8.9	3.1
Education		
BA/BS degree	48.6	43.0
Birthplace		
Foreign born	20.1	25.1
Marital Status		
Married	77.1	73.6
Single, never married	5.8	6.5
Other (widowed, separated, divorced, living with partner)	16.8	19.9
Body Mass Index		
0–18.49	0.6	0.6
18.5–24.99	25.2	28.0
25.0–29.99	46.8	48.0
30.0 or higher	27.2	23.4
PSA test ever	57.6	44.0

* Based on data from the California Health Interview Survey. Copyright ^© ^2003 The Regents of the University of California. All Rights Reserved.

**Table 7:** Percent agreement between BMI calculated from self-reported weight and height and from electronic clinical databases: CMHS Cohort^1^, California.

	Self-Reported BMI^2^			
BMI from clinical databases^2^	<25	25–29	30+	Total

<25	3059	596	34	3689
25–29	1078	6652	487	8217
30+	94	1424	5669	7187
				
Total	4231	8672	6190	19 093
				
% Agreement				80.6%

^1 ^BMI validation includes only participants from northern California who had electronic BMI data available

^2 ^BMI categories based on categories reported in clinical databases.
